# Do lifestyle, anthropometric and demographic factors associated with muscle strength differ in a UK and Japanese cohort? An exploratory analysis

**DOI:** 10.1007/s40520-023-02614-5

**Published:** 2023-11-10

**Authors:** Elaine M. Dennison, Faidra Laskou, Leo D. Westbury, Gregorio Bevilacqua, Nicholas R. Fuggle, Toshiko Iidaka, Chiaki Horii, Sakae Tanaka, Noriko Yoshimura, Cyrus Cooper

**Affiliations:** 1 MRC Lifecourse Epidemiology Centre, University of Southampton, Southampton General Hospital, Southampton, SO16 6YD UK; 2 Department of Preventive Medicine for Locomotive Organ Disorders, 22nd Medical and Research Center, University of Tokyo, Tokyo, Japan; 3 Department of Orthopaedic Surgery, University of Tokyo, Tokyo, Japan

**Keywords:** Lifestyle factors, Muscle weakness, Older adults, Grip strength

## Abstract

**Background:**

Muscle weakness is associated with adverse clinical outcomes including disability and mortality. We report demographic, anthropometric and lifestyle correlates of grip strength in UK and Japanese population-based cohorts.

**Aim:**

To report prevalence of low grip strength according to 2019 European Working Group on Sarcopenia in Older People (EWGSOP2) and 2019 Asian Working Group for Sarcopenia (AWGS 2019) thresholds and to consider correlates of grip strength in Eastern and Western populations.

**Methods:**

UK (1572 men; 1415 women) and Japanese (519 men; 1027 women) participants were recruited from two cohorts harmonised by consensus. Muscle strength was measured by grip strength dynamometry. Potential correlates of grip strength were examined using sex-stratified linear regression; univariate correlates (*p* < 0.05) were included in mutually adjusted models.

**Results:**

Mean (SD) age was 66.2 (2.8) and 65.8 (12.3) in UK and Japanese cohorts, respectively. Prevalence of low grip strength was higher in Japanese participants (EWGSOP2 5.4% versus 2.4%, AWGS 2019 9.0% versus 3.7%). In both cohorts and sexes, univariate correlates of lower grip strength were older age, shorter height, not consuming alcohol, leaving education earlier and greater comorbidity. Apart from older age and shorter height, the only factors related to lower grip strength in mutually adjusted analyses were greater comorbidity among UK participants (kg difference in grip strength (95%CI) per additional comorbidity − 0.60(− 0.98, − 0.21) among men and − 0.50(− 0.86, − 0.13) among women) and not consuming alcohol among Japanese men (− 1.33(− 2.51, − 0.15)).

**Discussion:**

Correlates of muscle strength were similar in both cohorts.

**Conclusions:**

A global approach to age-related muscle weakness prevention may be appropriate.

## Introduction

Grip strength is well recognised as an important predictor of adverse clinical outcomes, including disability and death [1]. Several studies have reported relationships of grip strength with comorbidity, specifically diabetes and mental health in both Western and Asian populations [2–5]. Most recently, work that considered relationships in the oldest old, the Leiden Study, reported relationships between functional and cognitive performance and age-related muscle weakness [6]. Hence, recognition of the presence of age-related muscle weakness, assessed by measuring grip strength with a handheld dynamometer, is important as it might be prevented by health interventions such as exercise, and might reduce the burden of clinical sequelae [7].

While previous studies have considered the lifestyle determinants of age-related muscle weakness, with much of the epidemiological data coming from Brazil and Asia [8–10], no previous work has directly compared findings in two comparable cohorts in contrasting geographic regions. From the published studies available [8–10], the condition is thought to be common, with community-based studies suggesting rates for the condition in men and women, respectively of 19% and 27% among individuals aged 60 to 69 years, 31% and 42% for those aged 70 to 79 years, and around 45% for those aged 80 years and above.

The aetiology of age-related muscle weakness is unknown but is thought to represent an interplay of genetic and environmental factors [6, 8]. We have previously reported differences in the prevalence of frailty, osteoarthritis and osteoporosis between the UK and Japan, highlighting possible differences in lifestyle between the two countries which might be relevant to the relative frequency of development of poor muscle strength in the two populations [11–13]. Given the relative paucity of available epidemiological data, and the clinical importance of its recognition, the aims of this study were to report the prevalence of muscle weakness using the 2019 European Working Group on Sarcopenia in Older People (EWGSOP2)[14] and 2019 Asian Working Group for Sarcopenia (AWGS 2019) grip strength thresholds [15] and examine demographic, anthropometric and lifestyle associations with grip strength as a continuous outcome among population-based cohorts in the UK and Japan.

## Methods

### Hertfordshire Cohort Study, UK

Individuals enrolled in the Hertfordshire Cohort Study were selected with the help of the National Health Service Central Registry at Southport, and Hertfordshire Family Health Service Association [16]. We traced 8650 men and women who were born between 1931 and 1939 in Hertfordshire, who still lived there during the period 1998–2004. Of these, 7106 were confirmed as registered with a Hertfordshire GP. We were given written permission from the General Practitioners of 6099 subjects to contact them. We approached each person by letter, asking them if they would be willing to be contacted by one of our research nurses. Three thousand two hundred and twenty five participants agreed to participate in a home interview with a trained research nurse, during which a structured questionnaire was administered. The details of cohort construction, methodology and confirmation of representation of the UK population have been published [16]. In brief, we administered a questionnaire that included information on socioeconomic status, medical history, cigarette smoking, alcohol consumption (frequency of drinking alcohol, type of alcohol consumed, number of units consumed each time), dietary calcium intake, comorbidities, SF-36 and reproductive variables in women. Physical activity was assessed by a previously validated questionnaire. Of these, 2997 (93%) then attended a clinic for detailed physiological investigations. Height was measured to the nearest 0.1 cm using a Harpenden pocket stadiometer (Chasmors Ltd, London, UK) and weight to the nearest 0.1 kg on a SECA floor scale (Chasmors Ltd, London, UK). Body mass index (BMI) was calculated as weight divided by height^2^ (kg/m^2^). Grip strength was measured three times on each side using a Jamar handgrip dynamometer; the highest of the six measurements was used for analysis. Clinics were provided in North, East and West Hertfordshire. The HCS baseline investigations had ethical approval from the Hertfordshire and Bedfordshire Local Research Ethics Committee and all subjects gave written informed consent.

### The ROAD study

The ROAD study started in 2005. It is a large-scale prospective study of musculoskeletal diseases that consists of population-based cohorts from several communities in Japan. Details of the cohort profiles have been reported elsewhere [17]. Briefly, between 2005 and 2007, a baseline database was created that included the clinical and genetic information of 3,040 residents. The subjects were recruited from resident registration listings in three communities with different characteristics: 1350 subjects from an urban region in Itabashi, Tokyo; 864 subjects from a mountainous region in Hidakagawa, Wakayama; and 826 subjects from a coastal region in Taiji, Wakayama. All participants provided written informed consent, and the study was approved by the ethics committees of the University of Tokyo (No. 1264 and No. 1326) and Wakayama Medical University (No. 373). Participants completed an interviewer-administered questionnaire that comprised questions related to lifestyle, including smoking habits, alcohol consumption (frequency of drinking alcohol, amount consumed each time), medical history, and health-related quality of life (QOL). Anthropometric measurements, including height and weight, were measured for all participants. The body mass index (BMI; weight [kg]/height^2^ [m]) was calculated. To assess muscle strength, handgrip strength was measured using a handgrip dynamometer (Toei Light Co., Ltd., Saitama, Japan). Both hands were tested, and the larger value was used to determine the maximum muscle strength.

### Statistical analyses

Questionnaire variables were harmonised through consensus at a meeting between the two Principal Investigators and subsequent meetings to review analysis. Number of comorbidities out of bronchitis, diabetes, ischaemic heart disease (IHD), hypertension and stroke were scored, with range 0–5 for participants in both cohorts. In both cohorts, continuous variables were normally distributed and, therefore, means and standard deviations were used as descriptive statistics. Categorical variables were expressed as frequencies and percentages. Participants were categorised as drinkers or non-drinkers based on whether they reported consuming any alcohol or not in their lifestyle questionnaire. Age-related muscle weakness was classified according to thresholds proposed by EWGSOP2 (< 27 kg in men, < 16 kg in women)[14] and AWGS 2019 [15] (< 28 kg in men, < 18 kg in women). Differences in characteristics between cohorts were examined using t-tests for continuous variables and chi-squared tests for categorical variables. In each cohort, cross-sectional associations between demographic, anthropometric and lifestyle characteristics and muscle strength were examined using sex-stratified univariate linear regression with grip strength considered as a continuous outcome. Multivariate analysis was performed by fitting a mutually adjusted model for each sex among each cohort; the mutually adjusted model comprised exposures that were significantly associated with the outcome in the corresponding univariate analysis. A *p*-value of < 0.05 was considered to be statistically significant. The analyses were conducted using Stata, version 14. The analysis sample comprised participants with grip strength values.

## Results

The characteristics of the UK and Japanese populations are shown in Table 1. Mean (SD) age was similar in the two cohorts; that of UK participants was 65.7 (2.9) years in men and 66.6 (2.7) years in women, while in Japan this was 66.6 (12.4) years in men and 65.5 (12.2) years in women. UK participants were noted to have a higher BMI, with UK participants typically around 10 cm taller than their Japanese counterparts, while Japanese men were the most likely to smoke (24.7% in contrast to 15.1% of UK men who were current smokers, while 3.9% of Japanese women and 9.8% of UK women were current smokers). In the UK, 19.4% of men and 17.2% of women left education before their 15^th^ birthday; in Japan the corresponding figures were 11.5% and 10.0%. Reporting drinking any alcohol was more common in the UK; in Japan only 21.5% of women reported drinking any alcohol, with the majority reporting drinking less than one unit each time, while in the UK the median alcohol consumption was 8.8 units per week in men and 1.5 units per week in women.

**Table 1:** Summary characteristics of study participants

Characteristic	Mean (SD) or *n* (%)						*P*-value ^a^
	Hertfordshire Cohort Study (UK)			ROAD study (Japan)			
	Men (*n* = 1572)	Women (*n* = 1415)	All (*n* = 2987)	Men (*n *= 519)	Women (*n* = 1027)	All (*n* = 1546)	
Age (years)	65.7 (2.9)	66.6 (2.7)	66.2 (2.8)	66.6 (12.4)	65.5 (12.2)	65.8 (12.3)	0.180
Height (cm)	174.2 (6.5)	160.8 (5.9)	167.8 (9.1)	164.1 (7.0)	151.1 (6.9)	155.5 (9.2)	< 0.001
BMI (kg/m^2^)	27.2 (3.8)	27.6 (4.9)	27.4 (4.4)	23.6 (3.4)	23.1 (3.5)	23.3 (3.5)	< 0.001
Current alcohol drinker	1483 (94.4%)	1132 (80.0%)	2615 (87.6%)	326 (62.8%)	221 (21.5%)	547 (35.4%)	< 0.001
Current smoker	238 (15.1%)	139 (9.8%)	377 (12.6%)	126 (24.7%)	40 (3.9%)	166 (10.9%)	0.085
Left education before age 15 years	304 (19.4%)	243 (17.2%)	547 (18.3%)	58 (11.5%)	101 (10.0%)	159 (10.5%)	< 0.001
Grip strength (kg)	44.0 (7.5)	26.5 (5.8)	35.7 (11.0)	40.0 (8.9)	25.6 (5.8)	30.4 (9.8)	< 0.001
Low grip strength (EWGSOP2)^b^	21 (1.3%)	50 (3.5%)	71 (2.4%)	35 (6.7%)	49 (4.8%)	84 (5.4%)	< 0.001
Low grip strength (AWGS 2019)^c^	26 (1.7%)	86 (6.1%)	112 (3.7%)	42 (8.1%)	97 (9.4%)	139 (9.0%)	< 0.001

*EWGSOP* European Working Group on Sarcopenia in Older People

*AWGS* Asian Working Group for Sarcopenia

^a^P-value for difference in the characteristic between the whole analysis sample of the Hertfordshire Cohort Study and the ROAD study; these were calculated from t-tests and chi-squared tests as appropriate

^b^ < 27 kg (men), < 16 kg (women)

^c^ < 28 kg (men), < 18 kg (women)

The prevalence of age-related muscle weakness in the UK was 1.3% in men and 3.5% in women using the EWGSOP2 grip strength thresholds (< 27 kg for men and < 16 kg for women); this was higher in Japan (6.7% in men, 4.8% in women) as shown in Table 1. Prevalence of muscle weakness was also higher in Japan using the AWGS 2019 thresholds (< 28 kg in men, < 18 kg in women) at 8.1% among men and 9.4% among women, compared to 1.7% among UK men and 6.1% among UK women. As shown above, a higher prevalence of muscle weakness was observed among women, compared to men, when the EWGSOP2 thresholds were implemented among UK participants and when the AWGS 2019 thresholds were implemented among Japanese participants.

Cross-sectional associations between participant characteristics and grip strength among UK participants are presented in Table 2. Among men and women, older age, shorter height, not currently consuming alcohol, leaving education earlier and greater numbers of comorbidities were related to lower grip strength in univariate analyses. In mutually adjusted analyses which simultaneously included these exposures, age (kg difference in grip strength (95% CI) per additional year of age: − 0.35 (− 0.47, − 0.22) among men and − 0.21 (− 0.32, − 0.11) among women), height (0.44 (0.39, 0.49) among men and 0.26 (0.21, 0.31) among women per cm increase in height) and comorbidity (− 0.60 (− 0.98, − 0.21) among men and − 0.50 (− 0.86, − 0.13) among women per additional comorbidity) remained statistically significant among men and women.

**Table 2:** Difference in grip strength (kg) according to each exposure among UK men and women in the Hertfordshire Cohort Study

Exposure	Men				Women			
	Univariate		Mutually adjusted		Univariate		Mutually adjusted	
	Estimate (95% CI)	*P*-value	Estimate (95% CI)	*P*-value	Estimate (95% CI)	*P*-value	Estimate (95% CI)	*P*-value
Age (years)	− 0.49 (− 0.61, − 0.36)	< 0.001	− 0.35 (− 0.47, − 0.22)	< 0.001	− 0.26 (− 0.37, − 0.15)	< 0.001	− 0.21 (− 0.32, − 0.11)	< 0.001
Height (cm)	0.47 (0.41, 0.52)	< 0.001	0.44 (0.39, 0.49)	< 0.001	0.27 (0.22, 0.32)	< 0.001	0.26 (0.21, 0.31)	< 0.001
Current smoker	− 0.53 (− 1.57, 0.51)	0.317			0.50 (− 0.51, 1.51)	0.333		
Current alcohol drinker	1.77 (0.15, 3.38)	0.032	0.76 (− 0.76, 2.28)	0.329	1.02 (0.27, 1.77)	0.008	0.60 (− 0.13, 1.34)	0.109
Age left education (years)	0.61 (0.35, 0.88)	< 0.001	0.23 (− 0.02, 0.48)	0.075	0.39 (0.16, 0.63)	0.001	0.18 (− 0.05, 0.41)	0.124
Comorbidity score*	− 0.94 (− 1.37, − 0.52)	< 0.001	− 0.60 (− 0.98, − 0.21)	0.002	− 0.73 (− 1.11, − 0.35)	< 0.001	− 0.50 (− 0.86, − 0.13)	0.008

CI Confidence interval

*Number of comorbidities out of bronchitis, diabetes, IHD, hypertension and stroke

Difference in grip strength is presented per unit increase in the exposure or for the presence versus absence of the exposure

For each sex, statistically significant associations in univariate analyses were included in a mutually adjusted model

Table 3 presents cross-sectional associations between participant characteristics and grip strength among Japanese participants. In univariate analyses, the same characteristics were associated with lower grip strength as in UK participants: older age, shorter height, not currently consuming alcohol, leaving education earlier and greater numbers of comorbidities. As in UK participants, age (− 0.36 (− 0.42, − 0.30) among men and − 0.22 (− 0.25, − 0.19) among women per additional year of age) and height (0.44 (0.35, 0.54) among men and 0.27 (0.21, 0.32) among women per cm increase in height) remained statistically significant among men and women in multivariate analyses; not currently consuming alcohol was only related to lower grip strength in multivariate analyses among men (1.33 (0.15, 2.51) for consuming alcohol as opposed to being a non-drinker).

**Table 3:** Difference in grip strength (kg) according to each exposure among Japanese men and women in the ROAD study

Exposure	Men				Women			
	Univariate		Mutually adjusted		Univariate		Mutually adjusted	
	Estimate (95% CI)	*P*-value	Estimate (95% CI)	*P*-value	Estimate (95% CI)	*P*-value	Estimate (95% CI)	*P*-value
Age (years)	− 0.48 (− 0.52, − 0.43)	< 0.001	− 0.36 (− 0.42, − 0.30)	< 0.001	− 0.30 (− 0.33, − 0.28)	< 0.001	− 0.22 (− 0.25, − 0.19)	< 0.001
Height (cm)	0.76 (0.67, 0.84)	< 0.001	0.44 (0.35, 0.54)	< 0.001	0.49 (0.45, 0.54)	< 0.001	0.27 (0.21, 0.32)	< 0.001
Current smoker	1.49 (− 0.29, 3.27)	0.101			0.92 (− 0.93, 2.77)	0.330		
Current alcohol drinker	3.93 (2.37, 5.48)	< 0.001	1.33 (0.15, 2.51)	0.027	2.22 (1.36, 3.09)	< 0.001	0.63 (− 0.09, 1.34)	0.087
Age left education (years)	0.36 (0.20, 0.52)	< 0.001	− 0.06 (− 0.17, 0.06)	0.357	0.50 (0.40, 0.60)	< 0.001	0.00 (− 0.10, 0.10)	0.972
Comorbidity score*	− 1.74 (− 2.88, − 0.59)	0.003	0.78 (− 0.05, 1.61)	0.067	− 1.52 (− 2.06, − 0.97)	< 0.001	0.13 (− 0.30, 0.57)	0.541

*CI* confidence interval

*Number of comorbidities out of bronchitis, diabetes, IHD, hypertension and stroke

Difference in grip strength is presented per unit increase in the exposure or for the presence versus absence of the exposure

For each sex, statistically significant associations in univariate analyses were included in a mutually-adjusted model

## Discussion

We have reported the prevalence of age-related muscle weakness in a Japanese and UK community-dwelling cohort according to AWGS 2019 and EWGSOP2 thresholds respectively, demonstrating a higher prevalence in women compared to men. Despite a different prevalence of age-related muscle weakness in comparable cohorts based in the UK and Japan, the anthropometric and lifestyle determinants of grip strength were very similar. In each cohort, older age, shorter height, not currently consuming alcohol, leaving education earlier and greater comorbidity were related to lower grip strength among men and women in each cohort when univariate analyses were performed. In multivariate analyses, older age and shorter height were related to lower grip strength among both sexes in each cohort; greater comorbidity was only related to lower grip strength in UK participants; and not currently consuming alcohol was only related to lower grip strength among Japanese men. The similarity of findings between the two cohorts suggests that a global approach to the prevention of muscle weakness may be appropriate.

There are, of course, several limitations to our study. Our study focuses on muscle strength as the key outcome, rather than muscle mass or performance, both of which are also closely associated with adverse clinical outcomes [1, 14]. The two cohorts were recruited independently, and while the sampling approach was similar at both sites and we harmonised the exposure variables considered, some methodological differences will remain. Specifically, the data collected regarding education in the two cohorts was very different, with far more detailed information available in Japan. Harmonisation meant we had to reduce the data available in both cohorts to the minimum common information, so we were unable to assess some lifestyle variables such as physical activity and diet. Alcohol consumption was recorded only as a dichotomous variable in Japan, but many Japanese cohort members reporting regular alcohol consumption, only typically drinking 1 unit of alcohol at a time. We presented figures for age-related muscle weakness and reported associations and relationships using grip strength as a continuous outcome as the prevalence of age-related muscle weakness was low in HCS. We used muscle strength rather than sarcopenia as an outcome as harmonisation to allow categorisation of sarcopenia status was not possible in the two cohorts. Of note, we have previously demonstrated higher rates of frailty in the UK compared to Japan [13], suggesting maintenance of physical measures may be more stable in the East versus the West—longitudinal studies are required to test this. Comorbidities were self-reported, and we have no information regarding duration of disease. Finally, a healthy responder bias has been observed in HCS [16] and this cohort comprised of only community-dwelling older people; this may limit the generalisability of findings to the wider population of UK men and women in this age range. However, HCS participants were found to be broadly comparable with those in the nationally representative Health Survey for England [16]. Care should also be taken when generalising findings from the ROAD study to the wider population; whilst mean BMI was similar to nationally representative data, the proportion of current smokers and current drinkers was lower in the ROAD study, and information available related to frequency of drinking rather than type of alcohol consumed each time [17].

The relationship between alcohol consumption and muscle health that we observed, suggesting a protective effect of alcohol consumption in our cohorts is interesting, and most likely reflects the low levels of alcohol intake in participants. Analogous to bone health, where modest alcohol consumption is also associated with better bone health [18], our observations may reflect direct biological associations or confounding by other lifestyle factors. While alcohol excess is commonly associated with sarcopenic obesity [19], recent analyses from the UK Biobank reported highest grip strength among participants with medium alcohol intakes, and adverse relationships with high levels of alcohol consumption [20]. With regard to possible biological mechanisms, it may be relevant that polyphenol resveratrol, an important constituent of red wine, but not white wine, beer, or spirits, has been demonstrated to elicit a broad spectrum of biological responses that may be beneficial for cardiovascular risk (and possibly also muscle health) through impact on vascular function [21]. This area seems an important one for future research. Rather than a biological explanation for the relationships observed, it is also possible that participants with underlying comorbidity may have stopped drinking alcohol, with a consequent apparent protective effect of alcohol consumption.

Another dietary factor that may be relevant for muscle strength is green tea. It has been suggested that green tea, rather than other types of tea, may be beneficial for muscle health [22]. In a recent review, Haramizu and colleagues suggested that green tea catechins may exert their benefit on skeletal muscle health by maintaining a dynamic balance between protein synthesis and degradation and boosting synthesis of mitochondrial energy metabolism [23]. Green tea consumption is very uncommon in the UK, unlike in Japan where most cohort members reported drinking at least one cup of green tea per day.

We observed striking relationships between lower educational attainment and lower grip strength in both cohorts in univariate analyses. This is consistent with previous work—in a study sited in Brazil, researchers reported that in their cohort, education was similarly important [24]—past medical history of smoking may be relevant, but further studies in other cohorts are required where further consideration of confounders is warranted. The limitations in our data harmonisation and the low prevalence of some lifestyle factors such as cigarette smoking meant that it was harder to examine relationships between these and muscle health. Specifically smoking was uncommon in women in either cohort, and where we did consider this we could not adjust for pack years. However, other studies have suggested that smoking history and schooling was associated with dynapenia [8]. It is possible that some lifestyle factors such as diet are confounded by educational attainment.

We saw associations between higher numbers of comorbidities and lower grip strength in the UK and Japanese participants in univariate analyses but associations were only robust in multivariate analyses among UK participants. This may reflect the greater number of UK participants living with chronic disease, for example in the UK the prevalence of diabetes in our population was 14.6%, while it was 10.3% in Japan, reflecting possible better disease prevention in Japan. A recent Japanese study reported that both type 1 and type 2 diabetes were associated with a higher prevalence of muscle weakness, with worse diabetic control apparently increasing this risk, suggesting that the prevalence and management of the medical condition, rather than a difference in pathophysiology, may explain the differences between the two cohorts [25]. Several other studies have suggested relationships between muscle weakness and individual comorbidities in many populations, including other groups of patients with diabetes [26, 27], heart failure [28], chronic obstructive pulmonary disease [29] and Parkinson’s disease [30]. Other cohort studies have reported associations between polypharmacy and grip strength [31]. In some, but not all medical conditions, the presence of a chronic inflammatory state may be an important aetiological factor [32].

## Conclusions

In a study of a UK and Japanese cohort, we have demonstrated greater prevalence of age-related muscle weakness in females. In multivariate analyses, older age and shorter height were related to lower grip strength in both cohorts and sexes; greater comorbidity was related to lower grip strength among UK participants; and not consuming any alcohol was only related to lower grip strength among Japanese men. Our findings highlight many similarities in the determinants of low grip strength in a Western and Eastern population of older adults, and reinforce the need for a global approach to prevention.

## Data Availability

Hertfordshire Cohort Study and the ROAD study data are accessible via collaboration. Initial enquires should be made to EMD and NY, respectively (Principal Investigators). Potential collaborators will be sent a collaborators’ pack and asked to submit a detailed study proposal to the Steering Group of each study.
